# Supplementary material for: A Novel Synthetic Antibody Library with Complementarity-Determining Region Diversities Designed for an Improved Amplification Profile
Source: Int J Mol Sci. 2022 Jun 2;23(11):6255. doi: 10.3390/ijms23116255 (PMC9181208; doi:10.3390/ijms23116255)
Supplement: Supplementary file 1 [file ijms-23-06255-s001.zip › ijms-1699363-supplementary.pdf]

**Table S1. Primer sets for the amplification of CDRs from the oligonucleotide mixture.**

| Product name | Templates | Primers | |
|---|---|---|---|
| CDR-H1 | Oligomix[a] | VH3-1-f | VH3-1-b |
| CDR-H2 | Oligomix | VH3-2-f | VH3-2-b |
| Oligo-H3 | Oligomix | lib-cdr-f | lib-cdr-b |
| CDR-H3(#1~#8) | H3(#1~#8)[b] | VH-3-f | VH-3-b |
| CDR-L1 | Oligomix | VL-1-f | VL-1-b |
| CDR-L2 | Oligomix | VL-2-f | VL-2-b |
| CDR-L3 | Oligomix | VL-3-f | VL-3-b |
| CDR-K1 | Oligomix | VK-1-f | VK-1-b |
| CDR-K2 | Oligomix | VK-2-f | VK-2-b |
| CDR-K3 | Oligomix | VK-3-f | VK-3-b |

[a] Oligonucleotide pool synthesized by parallel synthesis.

[b] CDR-H3 oligonucleotides separated by length (9~16 aa).

**Table S2. Primers for the amplification of the framework regions for single-CDR library construction.**

| Product name | Templates | Primers | |
|---|---|---|---|
| 1 | VHVL-pUC57[a] | pUC57-b | VH3-1-f-rc |
| 2 | VHVL-pUC57 | pUC57-b | VH3-2-f-rc |
| 3 | VHVL-pUC57 | pUC57-b | VH-3-rc-F |
| 4 | VHVL-pUC57 | pUC57-b | VL-1-f-rc |
| 5 | VHVL-pUC57 | pUC57-b | VL-2-f-rc |
| 6 | VHVL-pUC57 | pUC57-b | VL-3-f-rc |
| 7 | VHVK-pUC57[b] | pUC57-b | VK-1-f-rc |
| 8 | VHVK-pUC57 | pUC57-b | VK-2-f-rc |
| 9 | VHVK-pUC57 | pUC57-b | VK-3-f-rc |
| 10 | VHVL-pFcF[c] | VH3-1-b-rc | hCH2-b |
| 11 | VHVL-pFcF | VH3-2-b-rc | hCH2-b |
| 12 | VHVL-pFcF | VH-3-rc-B | hCH2-b |
| 13 | VHVL-pFcF | VL-1-b-rc | hCH2-b |
| 14 | VHVL-pFcF | VL-2-b-rc | hCH2-b |
| 15 | VHVL-pFcF | VL-3-b-rc | hCH2-b |
| 16 | VHVK-pFcF[d] | VK-1-b-rc | hCH2-b |
| 17 | VHVK-pFcF | VK-2-b-rc | hCH2-b |
| 18 | VHVK-pFcF | VK-3-b-rc | hCH2-b |

[a] Codon-optimized VH3-23/JH4-($G_4S$)$_3$-Vλ1-47/Jλ2 gene cloned in pUC57 vector.

[b] Codon-optimized VH3-23/JH4-($G_4S$)$_3$-Vκ3-20/Jκ1 gene cloned in pUC57 vector.

[c] Codon-optimized VH3-23/JH4-($G_4S$)$_3$-Vλ1-47/Jλ2 gene cloned in pFcF vector (a pcDNA3.1-derived vector with a pair of SfiI sites and a hIgG1 Fc tag).

[d] Codon-optimized VH3-23/JH4-($G_4S$)$_3$- Vκ3-20/Jκ1 gene cloned in pFcF vector (a pcDNA3.1-derived vector with a pair of SfiI sites and a hIgG1 Fc tag).

[a-d]Codon-optimized scFv genes were synthesized with asymmetric SfiI restriction sites compatible with pComb3X phagemid vector at both ends.

**Table S3. Assembly of single-CDR scFv libraries by overlap extension PCR.**

| Product name | Templates | Primers | |
|---|---|---|---|
| VHVL -H1 | 1 + CDR-H1 + 10 | pUC57-in-b | hCH2-in-b |
| VHVL -H2 | 2 + CDR-H2 + 11 | | |
| VHVL -H3 | 3 + CDR-H3 + 12 | | |
| VHVL -L1 | 4 + CDR-L1 + 13 | | |
| VHVL -L2 | 5 + CDR-L2 + 14 | | |
| VHVL -L3 | 6 + CDR-L3 + 15 | | |
| VHVK -K1 | 7 + CDR-K1 + 16 | | |
| VHVK -K2 | 8 + CDR-K2 + 17 | | |
| VHVK -K3 | 9 + CDR-K3 + 18 | | |

**Table S4. Amplification of the proofread CDR and adjoining framework regions by PCR.**

| Product name | Templates | Primers | |
|---|---|---|---|
| VH3-1 | VHVL -H1 | pC3x-f | VH3-1-b |
| VH3-2 | VHVL -H2 | VH3-1-b-rc | VH3-2-b |
| VH3-H3 | VHVL -H3 | VH3-2-b-rc | VH-3-b |
| VL-1 | VHVL -L1 | VH-3-rc-B | VL-2-f-rc |
| VL-2 | VHVL -L2 | VL-2-f | VL-3-f-rc |
| VL-3 | VHVL -L3 | VL-3-f | pC3x-b |
| VK-1 | VHVK -K1 | VH-3-rc-B | VK-2-f-rc |
| VK-2 | VHVK -K2 | VK-2-f | VK-3-f-rc |
| VK-3 | VHVK -K3 | VK-3-f | pC3x-b |

**Table S5. Primers for the assembly of VH, VL/VK and scFv by OE-PCR.**

| Product name | Templates | Primers | |
|---|---|---|---|
| *Primers for the assembly of VH, VL, or VK domains* | | | |
| VH3 | VH3-1 + VH3-2 + VH3-H3 | pC3x-f | VH-3-b |
| VL1 | VL-1 + VL-2 + VL-3 | VH-3-rc-B | pC3x-b |
| VK3 | VK-1 + VK-2 + VK-3 | VH-3-rc-B | pC3x-b |
| *Amplification of the final scFv gene* | | | |
| VH3VL1 | VH3 + VL1 | pC3x-f | pC3x-b |
| VH3VK3 | VH3 + VK3 | pC3x-f | pC3x-b |

**Table S6. Sequences of the primer used in this work.***

| Primer name | Sequence (5'→3') |
|---|---|
| hCH2-b | CTTGACCTCAGGGTCTTC |
| hCH2-in-b | CCA GGA GTT CAG GTG CTG |
| lib-cdr-b | CTGAGTCGATGACCTACG |
| lib-cdr-f | GTCAGTCACGCTCTAAGG |
| pC3x-b | AAC CAT CGA TAG CAG CAC CG |
| pC3x-f | GCA CGA CAG GTT TCC CGA C |
| pUC57-b | TTCGCCATTCAGGCTGCG |
| pUC57-in-b | GGA TGT GCT GCA AGG CGA |
| VH3-1-b | TTACCTGGTGCCTGTCTG |
| VH3-1-b-rc | CAGACAGGCACCAGGTAA |
| VH3-1-f | CTCCGGATTCACTTTCAGC |
| VH3-1-f-rc | GCTGAAAGTGAATCCGGAG |
| VH3-2-b | GCGTGAGATGGTGAAGCG |
| VH3-2-b-rc | CGCTTCACCATCTCACGC |
| VH3-2-f | GGACTGGAGTGGGTCTCT |
| VH3-2-f-rc | AGAGACCCACTCCAGTCC |
| VH-3-b | CAGAGTACCTTGTCCCCA |
| VH-3-f | CACTGCCGTGTATTACTGC |
| VH-3-rc-B | TGGGGACAAGGTACTCTG |
| VH-3-rc-F | GCAGTAATACACGGCAGTG |
| VK-1-b | CCAGGTTTCTGTTGGTACCA |
| VK-1-b-rc | TGGTACCAACAGAAACCTGG |
| VK-1-f | CGCGCAACTCTGTCTTGT |
| VK-1-f-rc | ACAAGACAGAGTTGCGCG |
| VK-2-b | GAACCTGTCTGGGATGCC |
| VK-2-b-rc | GGCATCCCAGACAGGTTC |
| VK-2-f | CCACGCCTGCTCATCTAT |
| VK-2-f-rc | ATAGATGAGCAGGCGTGG |
| VK-3-b | ACCTTCGTTCCCTGACCA |
| VK-3-b-rc | TGGTCAGGGAACGAAGGT |
| VK-3-f | GACTTCGCAGTTTACTATTGT |
| VK-3-f-rc | ACAATAGTAAACTGCGAAGTC |
| VL-1-b | CTGGGAGTTGCTGATACCA |
| VL-1-b-rc | TGGTATCAGCAACTCCCAG |
| VL-1-f | CGCGTCACCATCAGCTGC |
| VL-1-f-rc | GCAGCTGATGGTGACGCG |
| VL-2-b | AAAGCGATCAGGCACACC |
| VL-2-b-rc | GGTGTGCCTGATCGCTTT |

| | |
|---|---|
| VL-2-f | CTCCTAAGCTCCTGATTTAC |
| VL-2-f-rc | GTAAATCAGGAGCTTAGGAG |
| VL-3-b | AGTTTGGTCCCACCGCCG |
| VL-3-b-rc | CGGCGGTGGGACCAAACT |
| VL-3-f | CGAGGCTGACTATTACTGC |
| VL-3-f-rc | GCAGTAATAGTCAGCCTCG |

* Oligonucleotides were ≥ 85% pure after purification using Oligonucleotide Purification Cartridge

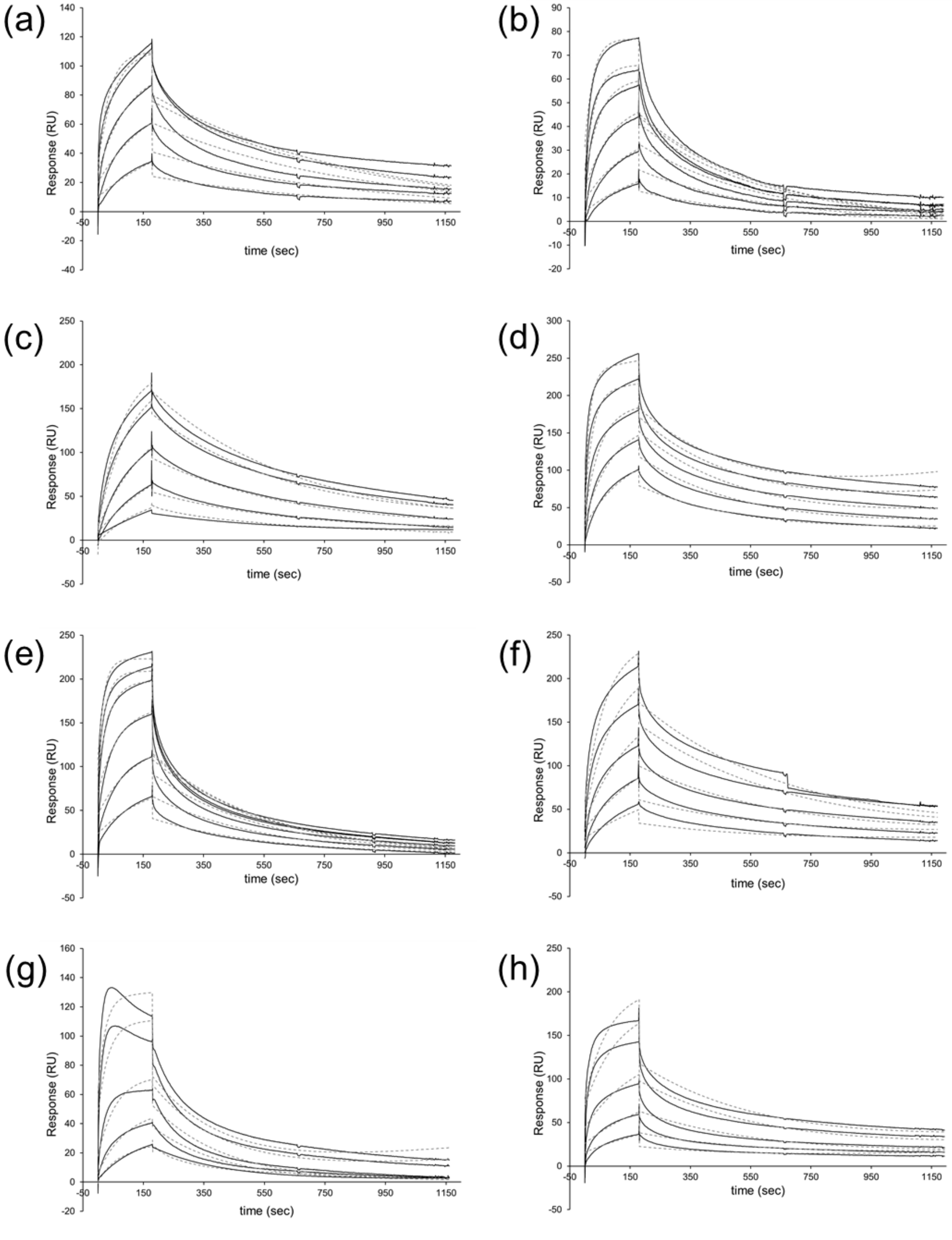
(a)
Response (RU)
time (sec)
(b)
Response (RU)
time (sec)
(c)
Response (RU)
time (sec)
(d)
Response (RU)
time (sec)
(e)
Response (RU)
time (sec)
(f)
Response (RU)
time (sec)
(g)
Response (RU)
time (sec)
(h)
Response (RU)
time (sec)

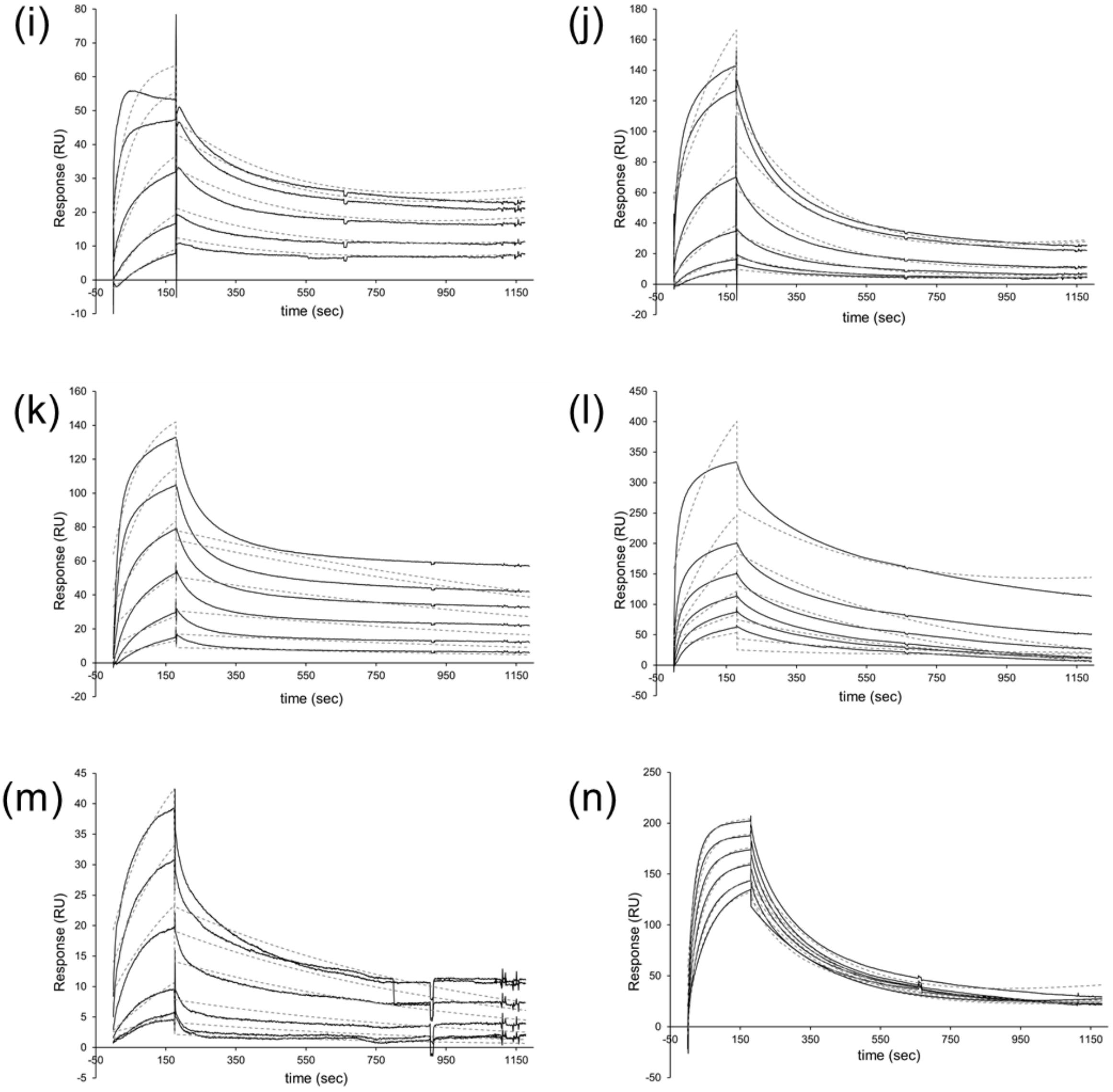


**Figure S1.** SPR sensorgrams (solid lines) and 1:1 Langmuir binding fitting (dashed lines) of OPAL-T clones. (a) AIMP1-C6 ([scFv] = 40–480 nM), (b) AIMP1-D4 ([scFv] = 67–1,070 nM), (c) AIMP1-E7 ([scFv] = 54–860 nM), (d) BCMA-A3 ([scFv] = 50–600 nM), (e) BCMA-B5 ([scFv] = 40–640 nM), (f) BCMA-D11 ([scFv] = 43–690 nM), (g) CARS-B6 ([scFv] = 31–375 nM), (h) CARS-D7 ([scFv] = 55–870 nM), (i) CDRS-D11 ([scFv] = 66–800 nM), (j) CARS-F4 ([scFv] = 5.7–91 nM), (k) CD22-D1 ([scFv] = 45–900 nM), (l) HEWL-H5 ([scFv] = 62–990 nM), (m) SARS-D6 ([scFv] = 5.7–91 nM), and (n) SARS-F4 ([scFv] = 80–230 nM).
